# Quality by Design Assisted Optimization and Risk Assessment of Black Cohosh Loaded Ethosomal Gel for Menopause: Investigating Different Formulation and Process Variables

**DOI:** 10.3390/pharmaceutics15020465

**Published:** 2023-01-31

**Authors:** Sradhanjali Mohapatra, Mohd. Aamir Mirza, Sayeed Ahmad, Uzma Farooq, Mohammad Javed Ansari, Kanchan Kohli, Zeenat Iqbal

**Affiliations:** 1 Nanotechnology Lab, School of Pharmaceutics Education and Research (SPER), Jamia Hamdard, New Delhi 110062, India; 2 Bioactive Natural Product Laboratory, Jamia Hamdard, New Delhi 110062, India; 3 Department of Pharmaceutics, College of Pharmacy, Prince Sattam Bin Abdulaziz University, Alkharj 16278, Saudi Arabia

**Keywords:** black cohosh, menopause, quality by design, risk assessment, QTTP, skin permeation, Ishikawa diagram, Pareto chart

## Abstract

Black cohosh (*Cimicifuga racemosa*) (CR) is a popular herb and is medically lauded for ameliorating myriad symptoms associated with menopause. However, its pharmaceutical limitations and non-availability of a patient-compliant drug delivery approach have precluded its prevalent use. Henceforth, the current research premise is aimed at developing an ethosomal gel incorporating triterpene enriched fraction (TEF) obtained from CR and evaluating its effectiveness through the transdermal application. TEF-loaded ethosomes were formulated using solvent injection, optimized and characterised. The optimized ethosomes were then dispersed into a polymeric gel base to form ethosomal gel which was further compared with the conventional gel by in-vitro and ex-vivo experiments. Here, the quality by design (QbD) approach was exploited for the optimization and development of ethosomal gel. The elements of QbD comprising initial risk assessment, design of experimentation (DoE), and model validation for the development of formulation have all been described in detail. The optimized ethosomes (F03) showed a nanometric size range, negative zeta potential and good entrapment. The in vitro release profile of gel revealed a burst release pattern following the Korsmeyer Peppas model having Fickian diffusion. The transdermal flux of ethosomal gel was observed to be more than that of conventional gel. Texture analysis and rheological characterization of the gel, revealed good strength showing shear thinning and pseudoplastic behaviour. The confocal microscope investigation revealed the deeper skin permeation of ethosomal gel than conventional gel. This result was further strengthened by DSC, IR and histological assessment of the animal skin (Wistar rat), treated with the optimized formulation. Conclusively, the implementation of QbD in the formulation resulted in a better understanding of the process and the product. It aids in the reduction of product variability and defects, hence improving product development efficiencies. Additionally, the ethosomal gel was found to be a more effective and successful carrier for TEF than the conventional gel through the transdermal route. Moreover, this demands an appropriate animal study, which is underway, for a stronger outcome.

## 1. Introduction

Menopause, a physiological arrest of menstruation is an essential trait in females which manifests into a spectrum of physically and mentally debilitating symptoms in the ageing population across the globe. Menopause-related syndrome often lasts for a few years and is associated with issues like hot flashes, night sweats, insomnia, dry vagina, mood swings, mental confusion, difficulty in concentrating and even depressive disorders [1,2]. Although, there is easy availability of pharmacological interventions like hormone replacement therapy (HRT) and other suitable agents to manage associated symptoms of menopause, but there is substantial apprehension in subjects for its usage due to adverse effects linked with long-term therapy. Alternatively, phytoestrogens, which majorly mimic the estrogenic effect can be consumed but albeit with troublesome effects on prolonged use [3]. Furthermore, botanicals when consumed orally may pose an unnecessary burden on the liver and could be detrimental to health [4,5,6]. One of the reason for this negative therapeutic outcome could be attributed to the presence of adulterants in herbal products that may negatively hamper health through this route [7,8].

To circumvent these unwanted adverse effects, transdermal drug delivery systems (TDDS) have proved to be a significant tool and may provide a possible solution. TDDS is a well-established system for delivering the drug to blood through the skin and offers several benefits in modern drug therapy. Such a type of delivery system is formulated to improve the plasma concentration of the drug with a lesser dose and dose frequency which ultimately enhances patient compliance by minimizing adverse effects. Moreover, it bypasses the biological shortcomings of the drugs like hepatic metabolism, liver toxicity, systemic side effects and other pharmaceutical caveats. These effects may be attributed to the reduction in dose through this route as compared to the conventional oral route [9]. Ethosomes have been reported to be novel, soft, flexible, non-invasive vesicles which are designed to deliver active moiety to the deeper skin levels and/or systemic circulation [10]. The uniqueness of ethosomes lies in the simplicity of their preparation, safety, and efficacy. These are comprised of phospholipid bilayers with ethanol as a vital adjuvant that makes it an impressive carrier to enhance skin permeability with incorporated drugs. Because of the distinct combination of lipids and ethanol, these are able to incorporate and deliver highly lipophilic moieties like testosterone, cannabinoids, erythromycin, and proteins etc. [11,12,13] across the most resistible stratum corneum layer of the skin. Therefore, this type of delivery system including the one used for herbal extract is expected to avoid complications arising due to shortcomings of oral herbal formulations and is seemingly a better choice for chronic uses through the skin.

Black cohosh/*Cimicifuga racemosa* (CR) is a well-established plant for lessening menopausal and post-menopausal syndrome through the oral route and is also available commercially in many countries such as the USA, Japan and Germany etc. [14]. The triterpenes which can be isolated from the root extract of the plant by fractionating with ethyl acetate is claimed to be the major constituent of CR and are responsible to alleviate menopause-related syndrome [15]. Therefore, a study was designed to fabricate a novel ethosomal gel incorporating the triterpene enriched fraction (TEF) to target systemic circulation through the skin. This formulation would expectably increase patient compliance by reducing the dose of the extract as well as associated side effects contrary to oral formulations of CR. For the very first time, this research tries to evaluate the effectiveness of CR gel for menopause through the transdermal route.

The ethosomal gel was optimized by utilizing quality by design (QbD) which comprises initial risk assessment, design of experimentation (DoE), and model validation. It also includes the determination of the quality target product profile (QTPP) using Failure Mode Effect Analysis (FMEA) for each quality attribute (QA) of the product. However, QTPP-based risk assessment necessitates the evaluation of the impact of material attributes and process parameters on the critical quality attributes (CQAs), which are described by the Ishikawa diagram [16,17]. Finally, central composite design (CCD) was employed to determine optimized formulation. Owing to the exploitation of QbD (systematic approach) for formulation development, unique benefits such as improved product and process understanding, process flexibility within the design space, implementation of more effective control strategies, easy scale-up, and a more robust product were achieved. The current work will assist the formulators as well as readers in understanding the fundamentals of using QbD in the design and development of a formulation. Additionally, this study tries to compare different attributes of the TEF-loaded ethosomal gel with a conventional gel to evaluate their superiority through the skin barrier.

## 2. Materials and Methods

### 2.1. Material

The plant material (Root powder) was procured from Navchetana Kendra, New Delhi, India (Batch No-BCE/17-18/967A) and was authenticated according to the protocol of WHO guideline [18] for powdered microscopy, physicochemical constraints and for presence/absence of contaminants. It was found in order at the Bioactive Natural Product Laboratory (BNPL), Department of Pharmacognosy and Phytochemistry, Jamia Hamdard, New Delhi, India. The voucher specimen (Voucher No. BNPL/JH/PhD/07/2018/02) has been deposited in the Laboratory, for future reference. TEF was prepared from the ethyl acetate fraction of the powdered root of the CR. Actein was purchased from Sigma Aldrich, Bangalore, India (Batch no-78877715). Lipoid S 100 was a kind gift from Lipoid, Germany. Triethanolamine was obtained from S.D Fine Chemicals Limited, Mumbai, India. Carbopol^®^ 971P NF was purchased from Lubrizol Advanced Materials, Inc. Tween 80 from Merck, Mumbai, India. Ethanol was purchased from Merck, Mumbai, India. Cellulose dialysis bag 12 KD was purchased from Sigma Aldrich, Bangalore, India. Nylon membrane filter (0.45 μm) was acquired MF-Millipore TM Membrane Filter, centrifugal filter devices (molecular weight cutoff 3000 daltons; Centricorn, Millipore, Bedford, MA, USA). The deionised and filtered water was used all over the study. All chemicals used throughout the experimentation were of analytical grade.

### 2.2. Preparations of TEF

200 g of the powdered root of the CR was accurately weighed and macerated overnight in 80% alcohol before being sonicated for 30 min. The filtrate was evaporated to dryness under reduced pressure. After drying, the extracted residue was dissolved in water and partitioned with the same volume of ethyl acetate thrice. The ethyl acetate portion contains the triterpenes which was then evaporated, concentrated under vacuum and labelled as TEF. Further, identified and confirmed by Liebermann Burchard test [19,20]. The extractive values were then calculated and stored at 4 °C.

Extractive value = (Weight of dried extract × 100)/Weight of plant material

### 2.3. Preparation of TEF-Loaded Ethosomes

The solvent injection method was used to prepare the ethosomal suspension as reported in the literature [21] with small alterations. In short, TEF (5 mg), lipoid S 100 (2% *w*/*v*), and tween 80 (1% *w*/*v*) were solubilized in ethanol (45% *v*/*v*) constituting the lipid phase and the container having the lipid phase was covered to circumvent ethanol evaporation on a magnetic stirrer with continuous stirring. The lipid phase was added drop by drop into the aqueous phase (water) (55% *v*/*v*) using a syringe (1 mL capacity, 24 gauge needle size) at a constant flow rate of 1 mL/min at a temperature of 30 °C with constant stirring. The suspension was continuously stirred on a magnetic stirrer (Remi Instrument, Mumbai, India) at 2300 rpm for additional 15 min to produce 1 mL of nano-size suspension. Later, the ethanol was evaporated to obtain TEF-loaded ethosomes and left undisturbed to cool at room temperature. Formulations were thereafter stored in the refrigerator (at 4 °C) and examined for vesicular shape, vesicle size, surface morphology, entrapment efficiency, in vitro drug permeation and stability.

### 2.4. Optimization and Development of TEF-Loaded Ethosomes Using the QbD Approach

The QbD technique was exploited for the optimization of the formulation which comprises three key stages such as initial risk assessment, DoE, and model validation. For the preliminary risk assessment, QTPP and CQAs were employed to study the effect of the concentration of ethanol, the concentration of lipid (lipoid S 100), the concentration of surfactant (tween 80), stirring time, stirring speed, pH, temperature, solubility, viscosity, needle type, injection rate and their consequence on particle size (or vesicular size) and their uniformity (through PDI), drug entrapment and drug release were premeditated. These outcomes were supported by the Pareto chart, a commonly used cause analysis tool and considered one of the ancillary quality tools in QbD [22]. QTPP of the final product was assessed and based on which dependent and independent factors were determined. Particle size, PDI and drug entrapment of the formulation were chosen as dependent factors. Further, independent factors such as the concentration of ethanol, and the concentration of lipids are found to be the critical parameters that can influence the CQAs of the final formulation [23].

CCD was utilized to formulate 13 combinations of independent factors and then the corresponding effect on dependent variables was noted and fed into the Design Expert version 11 by State Ease software to develop a design model or design space that can precisely predict an optimized formulation. Finally, an optimized formulation suggested by CCD was developed.

### 2.5. Vesicle Size, Polydispersity Index (PDI)

Dynamic light scattering (DLS) was employed at room temperature to determine the average particle size, particle size distribution, and surface charge of the formulated ethosomes using a Malvern Zeta sizer Nano ZS (Malvern Instruments, UK). The ethosomal formulation was dispersed in HPLC water with an instrument set at a 90° angle, a medium viscosity of 0.8862 cp, and a refractive index of 1.361. The particle size distribution and PDI were observed, and all measurements were done in triplicate [24].

### 2.6. Entrapment Efficiency

The entrapment efficiency of the ethosomal suspension was determined by using the ultrafiltration method using centrifugal filter devices (molecular weight cutoff 3000 daltons; Centricorn, Millipore, Bedford, MA, USA). The donor chamber was filled with ethosomal suspension (1 mL), and the unit was centrifuged at 11,000 rpm for 60 min. The encapsulated TEF present in the sample remained in the donor compartment (upper), while the unentrapped portion was collected in the lower sample recovery compartment across the filter. The concentration of the entrapped and unentrapped TEF (active constituents here) was estimated using a UV spectrophotometer (UV-1601/Shimadzu Corp, Kyoto, Japan), the total triterpene in TEF was quantified with respect to the standard marker actein at λmax 212 nm. Measurements were made in triplicates [25,26,27].

The following formula was used to calculate the entrapment efficiency

Entrapment efficiency = (T − C)/T × 100

where ‘T’ is the total amount of TEF and ‘C’ is the amount of TEF detected in the Supernatant.

### 2.7. Characterization of Optimized Ethosomal Formulation

#### 2.7.1. Morphology of the Ethosomes by Transmission Electron Microscopy (TEM)

TEM (AIIMS, Delhi) at 200 kV and 9900× magnification was used to examine the morphology of prepared ethosomes. About 20 μL of ethosomal suspension was suitably diluted with Millipore water and stained for about 30 s with 2% *w*/*v* phosphotungstic acid. The sample was dried on a coated copper grid. For each sample, two grids were created and viewed at random.

#### 2.7.2. Zeta Potential

Malvern Zetasizer (Nano-ZS, Malvern, UK) was used to determine the zeta potential of optimized formulation. The ethosomal suspension was first diluted with HPLC water and then the sample was filled in a sample cell up to the mark and analysed, all measurements were done in triplicate [28].

### 2.8. Stability Studies

Being an essential parameter storage stability of ethosomal suspension was measured by storing it at 4 °C ± 0.5 °C for a period of 4 weeks. The stability was then expressed with respect to particle size variation, PDI and drug entrapment. Measurements were done in triplicate at an interval of 0, 1, 2, 3 and 4 weeks.

### 2.9. Fabrication of TEF-Loaded Ethosomal Gel

**Figure 1:**
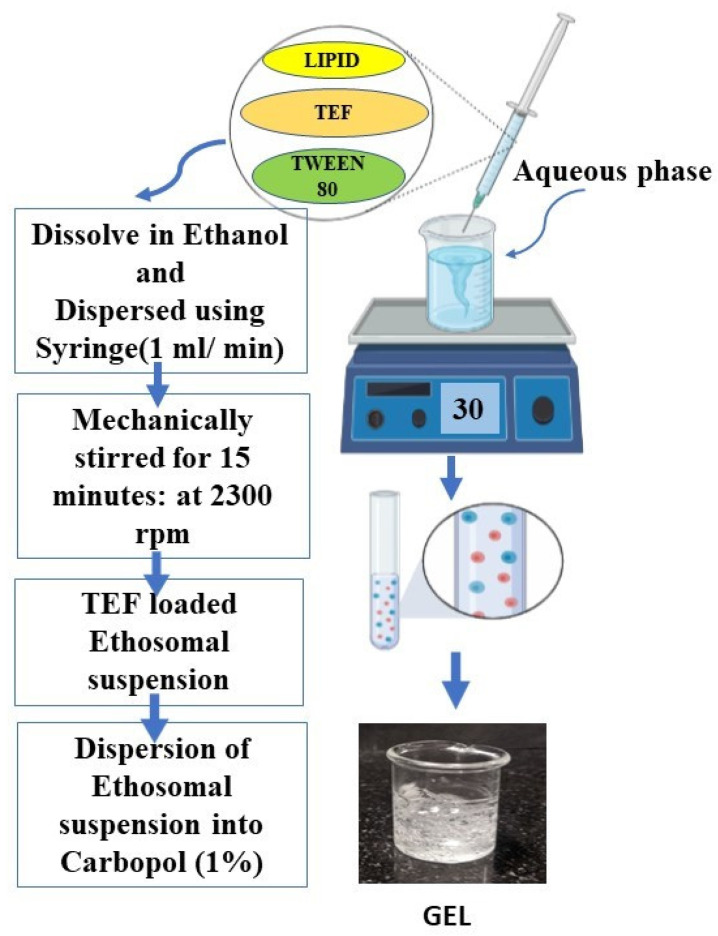
Fabrication methodology for the ethosomal gel incorporating TEF. The solvent injection method was used to prepare the ethosomal suspension which was then suitably dispersed in the carbopol to form TEF-loaded ethosomal gel.

Carbopol^®^ 971P NF (1% *w*/*v*) was solubilized in the requisite volume of water for about 6 h and later sonicated for 10 min to remove air bubbles. Ethosomal suspension comprising TEF was added dropwise to the previously swollen polymer with continuous stirring. Stirring was continued in an uncovered container at a speed of 700 rpm at room temperature to achieve a homogeneous ethosomal gel having an adequate consistency without lumps. The addition of triethanolamine (TEA) was carried out to neutralize and adjust the pH 7 with slow stirring until a clear transparent gel was achieved [29,30]. Figure 1 depicts the method of preparing TEF-loaded ethosomal suspension and ethosomal gel in the pictorial form [31,32]. Furthermore, preliminary risk assessment, QTPP and CQAs were employed to study the effect of the viscosity, homogeneity, content uniformity and release etc. in ethosomal gel.

### 2.10. Fabrication of Conventional TEF-Loaded Gel

The conventional gel of TEF was prepared by a similar method as that used for preparing the ethosomal gel previously described. In this method, the required quantity of TEF was taken and mixed into the gel base (same as that of the above) spiked with 2% Tween 80. The final concentration of TEF in the gel was fixed at 5 mg/mL.

### 2.11. Evaluation of TEF-Loaded Ethosomal Gel

#### 2.11.1. Homogeneity

The homogeneity was assessed by physically examining the gel.

#### 2.11.2. Drug Content

10 mL of ethanol was added to 1 g of TEF-loaded ethosomal gel and stirred continuously for 30 min. After centrifuging the mixture for 30 min at 3000 rpm, the supernatant was collected and passed through a membrane filter (pore size 0.45 μm). A UV spectrophotometer with a suitable dilution of samples was employed for analysis in triplicate [26,33].

Drug content (%) = TEF present in gel/Actual amount of TEF taken for gel × 100

#### 2.11.3. Rheological Characterization

The gels’ rheological properties were determined using a controlled stress rheometer (Physica MCR 101 Anton Parr Rheometer). The mechanical properties of both gels were investigated using oscillating tests and flow tests. Mechanical spectra as generated by the rheometer were captured at a frequency scale of 0.01–10 Hz. The viscoelastic region was evaluated, at 1 Hz, by stress sweep experiments. All samples were analyzed at 25 °C ± 1 °C with spindle number CP50 [34].

#### 2.11.4. Texture Analysis

The texture properties of the gel were determined using a Texture Analyzer TA. XT-Plus (Stable Micro Systems Ltd., Surrey, UK). A standard beaker (100 mL) was filled with approximately 50 mL of the gel formulation, avoiding the introduction of air into the sample and ensuring the generation of a smooth upper surface. A disc 40 mm in diameter was compressed into the gel and redrawn. The method settings, involving speed rate and distance (depth of insertion), were chosen based on the type of hydrogel. For each formulation, three replicate analyses were performed at room temperature, with the same conditions for each measurement. The force-time plot was used to calculate gel parameters such as firmness, consistency, cohesiveness, and work of cohesion (as depicted in Section 3.10). The maximum force represents the hardness/firmness of the hydrogel formulation (the maximum positive force required to achieve a given deformation, Fmax). However, cohesiveness is described as the amount of work required to deform the hydrogel during the probe’s downward movement (the negative area under the force-time curve: characterizes the work required to pull the probe away from the sample).

### 2.12. In Vitro Drug Release Study

The dialysis tube diffusion method was used to study the in vitro release of entrapped TEF from the conventional gel and ethosomal gel formulations. A weighed amount of conventional gel and ethosomal gel with an equivalent amount of TEF were separately kept in a dialysis membrane (MWCO10-12 kDa Himedia, India) and properly tied at both ends before being placed in separate beakers containing a 20 mL solvent mixture of 80% (*v*/*v*) PBS (pH 7.4) and methanol. The beakers were kept on magnetic stirrers with unremitting stirring at 100 rpm and a constant temperature, of 37 °C ± 1 °C. The sink conditions were maintained throughout the study. 1 mL of sample was withdrawn at 0.25, 0.5, 1, 2, 4, 6, 7.5, 10.5, 12, 18, 24, and 28 h, and was replaced by an equal amount of solvent blend in similar containers maintained at the pre-decided temperature. Samples were then analysed using the UV spectrometer to quantify the TEF as described in Section 2.6. Furthermore, release kinetic modelling was performed by plugging in vitro release data into various kinetic models such as zero-order, first-order, Higuchi, and Korsmeyer-Peppas [35]. The best fit model was determined by the model with the highest correlation coefficient value [36,37].

### 2.13. Ex Vivo Skin Permeation Study

Female Wistar rats (150–200 g) were used in the ex-vivo skin permeation study. Hair was removed from the dorsal region of rats in an area of 2 cm^2^ of skin with hair removal cream (Anne French; Wyeth Ltd., Hyderabad, India). The animals were sacrificed and the required skin was removed in order to obtain the hairless skin portion. After carefully removing the subcutaneous fat and connective tissues with a sharp scissor, the skin samples were washed with physiological salt solution (PSS) and distilled water before being stored in a refrigerator in PBS at 4 °C for later use. The ex vivo permeation from TEF-loaded ethosomal formulation through rat skin was determined using Franz’s diffusion cell, which was considered a semipermeable membrane. The skin was carefully clamped with Franz’s diffusion cell, which had an effective permeation area of 0.785 cm^2^ and a receptor volume of 10 mL, using phosphate buffer saline (pH 7.4). 1 g of the accurately weighed TEF-loaded ethosomal gel was transferred to the donor compartment of Franz’s diffusion cell and kept at 37 °C ± 1 °C with continuous stirring at 200 rpm by means of a magnetic stirrer. While the sink condition was maintained, samples were withdrawn at pre-set time periods (0.25, 0.5, 1, 2, 4, 6, 8 h.) and replaced with the same volume of blank receptor fluid in the receptor compartment. The same procedure was followed for TEF-loaded conventional gel. The amount of TEF released was measured spectrophotometrically as described in Section 2.6. The flux across the skin was calculated by dividing the slope of the cumulative amount of TEF permeated through the skin with time. Additionally, the permeation coefficients for gel were calculated by dividing flux with the total amount of TEF in the donor apartment. The experiment was carried out in triplicate and the results were reported as the mean ± SD [17]. All trials were made in agreement with the standard institutional guiding principles duly proved by the Committee for the Purpose of Control and Supervision of Experiments on Animals (CPCSEA) at Jamia Hamdard Animal Centre (New Delhi, India).

### 2.14. Skin Permeation Dynamics

Following the ex vivo skin permeation experiments, the ethosomal gel-treated skin was removed to ascertain the improvement of permeation across the skin using differential scanning calorimetry (DSC) and Fourier transform infrared (FTIR) in comparison to untreated skin as control. This experiment was approved by the Jamia Hamdard Animal Ethics Committee (JHAEC, Registration No. 1553/GO/ReBi/S/2000/CPCSEA), New Delhi, India.

#### 2.14.1. DSC Analysis of Skin

Both treated (ethosomal gel) and untreated skin were rinsed with PBS (pH 7.4), dried, and hermetically sealed in pans made of aluminium. DSC thermograms of treated and untreated skin were recorded using DSC (Perkin Elmer Inc., Waltham, MA, USA) at a scanning rate of 10 °C/min over a temperature range of 30–300 °C [34].

#### 2.14.2. FTIR Analysis of Skin

Ethosomal gel-treated skin was retrieved from the Franz diffusion cell, cut, washed with PBS (pH 7.4), dried and grounded into powder. The spectra of skins were then noted in the 500–4000 cm^−1^ range using FTIR (Perkin Elmer, Germany). Here, the spectra of untreated skin were taken as control which was prepared in the same way as that of treated skin [34].

#### 2.14.3. Histological Investigation of the Skin

Exposed hair removed Wistar rat skins were treated with the 230 mg of ethosomal gel (equivalent to 1 mg of TEF). The treated rats were sacrificed after 6 h, and skin samples were collected as described in the ex vivo skin permeation study. As a control, skin samples were taken from untreated rats. For 12 h, the skin samples were stored in 10% formalin solutions. The fixed samples were then dehydrated in ethanol and fixed in paraffin. The dyes (hematoxylin and eosin) were applied to the paraffin sections, which were then examined under an inverted light microscope (Olympus ix71, Olympus Corporation, Tokyo, Japan) [38].

### 2.15. Assessment of Depth of Skin Permeation

Confocal laser scanning microscopy 410 (Zeiss, Heidelberg, Germany) (CLSM) was used to detect and visualize the depth of permeation of rhodamine B solution, conventional gel and ethosomal gel across the skin. The skins were mounted on the Franz diffusion cells and treated with rhodamine B solution (0.5 mg/mL), rhodamine B-loaded conventional gel (0.5 mg/g gel) and rhodamine B-loaded ethosomal gel (0.5 mg/g gel) respectively to assess skin permeation. After 8 h, the treated skins were removed and cleaned with ethanol to remove any adhered formulation remnants. Then the fluorescence intensity were measured using CLSM after that they were vertically cut into pieces with 52 µm thickness and fixed them on the slides [39].

### 2.16. Skin Irritation Studies

To ascertain the toxicity of the gel, a skin irritation test was performed. Wistar rats were used in this study to investigate the irritancy of TEF solution and optimised TEF-loaded ethosomal gel using patch testing on the animal’s intact skin. The study protocol was approved by the Jamia Hamdard Animal Ethics Committee (JHAEC, Registration No. 1553/GO/ReBi/S/2000/CPCSEA), New Delhi, India. Animals with cleaned dorsal hair (1 cm^2^) were divided into three groups (1st group- control, 2nd group- TEF solution (hydro-alcoholic), 3rd group- optimized ethosomal gel) having 3 animals (*n* = 3) in each group housed under typical laboratory specifications (25 °C ± 1 °C and RH 55% ± 5%). Furthermore, 1 mg/mL of TEF solution and 230 mg of optimised TEF-loaded ethosomal gel (equivalent to 1 mg of TEF) were evenly applied to the clean-shaven skin of a rat three times in a day in three divided dose and observed for any obvious changes at 24, 48, and 72 h before being scored. A scale with a range of 0 to 4 was used to score the degree of erythema, with corresponding weightages of 0, 1, 2, 3, and 4 for no, slight, moderate, moderate to severe, and severe erythema, respectively.

## 3. Result and Discussion

### 3.1. Extractive Value

The extractive value of the plant material was found to be 10.2% ± 1.2% with total TEF content 2.5% ± 0.4%.

### 3.2. Preparation of TEF-Loaded Ethosomal Gel

As previously stated, the ethosomal suspension was prepared by the solvent injection technique by utilizing ethanol. The ethosomes were prepared using Lipoid S100 having a negative surface charge. As per the research conducted [40], the solvent injection method results in smaller-sized particles with relatively higher drug entrapment efficiencies. The prepared ethosomal suspension was then properly mixed with the polymeric solution to form a homogenous gel [23].

### 3.3. Optimization and Development of TEF-Loaded Ethosomal Gel

The ethosomal gel was prepared and was optimized by QbD based approach. Determination of the QTPP of the final product is the preliminary step to perform the QbD-based optimization. QTPP describes the CQAs that specify the desired quality standards along with the determination of the failure modes, reasons and risk quantification by using Failure Mode Effect Analysis (FMEA) for every quality attribute of the product. These are the fundamental requirements for a formulation. The elements of QTPP for quality characteristics of the formulation are described in Table 1 which further identifies the CQAs.

The sorting of CQAs from QTPP was based on failure mode analysis. The failure mode is related to the material attributes and process parameters of the formulation. The critical material attributes (CMAs) and the critical process parameters (CPPs) are likely to cause variations in CQAs. Therefore, the measurement of the impact of CMAs and CPPs on CQA and identification of their failure modes were measured using risk assessment as described in Table S1.

**Figure 2:**
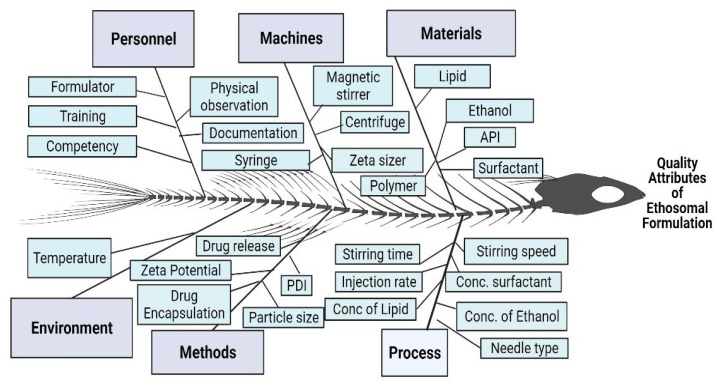
Ishikawa diagram explaining the key elements responsible for the quality attributes of the ethosomal gel.

QTPP-based risk assessment, involves the evaluation of the effect of material attributes and process parameters on the CQAs, as explained in the Ishikawa diagram (Figure 2). However, not all QAs need to have an equal impact on the QTPPs. Therefore, the impact of QAs on QTPP can be estimated through the FMEA (Table S2) where risk factors were quantified [37]. Here, each attribute/parameter (associated with both CMAs and CPPs) of failure modes was analysed on the basis of their failure effect, potential cause and control established on the formulation. Subsequently, potential cause and their probable incidence for the identified parameters were valued. A generous effort towards the establishment of the possible controls, inherent process and material-based mechanism was explored that could exert the early detection of faults. Accordingly, the quotient of severity (S), probability of occurrence (O)and probability of detectability (D) was quantified using scores as mentioned in the scoring table (Table S3). These scores were designed by the formulator considering S, O and D of the CQAs associated with the formulation. Based on the scores of S, O and D, the risk was quantified using prioritized numbers, that is the product of S, O and D. This is commonly denoted as risk priority number (RPN). The rationale is mentioned in Table S4 (Risk-level rating criteria), where the outcome of any alteration of process parameters/attributes of material is indicated as a potential risk. These risks were then denoted as low, medium and high accordingly. Table S4 gave a realistic picture with a correlation of all variables related to process and materials with scrupulous importance to CQAs resulting in precise quantification of threat during the formulation process [41].

**Figure 3:**
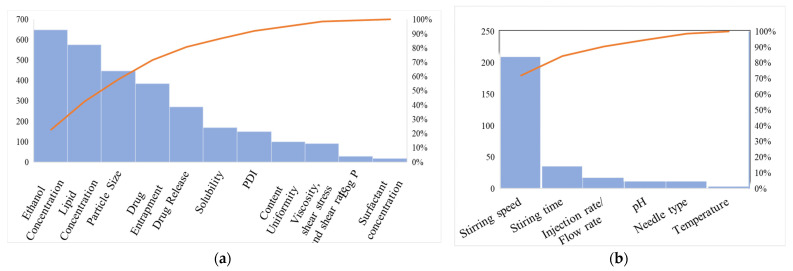
Pareto chart explaining quality attributes (**a**) critical material attributes (**b**) critical process parameters.

The extent of contributions of each quantified threat was denoted using a Pareto chart. Their contributions were segregated by their influence in CMAs and CPPs. In other words, it is a graphical representation that divides material/process problems from most common to least common. A typical Pareto chart with respect to CMAs and CPPs is depicted separately in (Figure 3a,b) [41]. It aids in the identification and determination of the root causes of problems during formulation.

For optimization of ethosome, an initial risk assessment was performed wherein the effect of lipid concentration, ethanol concentration, surfactant concentration, temperature, stirring time, stirring speed and their effect on particle size and PDI were observed (Table S5). Based on these outcomes, process variables such as time of stirring, speed of stirring, temperature and material attribute such as surfactant concentration were fixed as shown in Table 2. The remaining variables such as lipid concentration and ethanol concentration were treated as independent variables in the CCD and their influence on particle size and particle entrapment efficiency (dependent variables) was analysed via 22-level factorial design. Table 3 shows the various factors and their ranges in Design Expert software with constraints for dependent variables.

**Figure 4:**
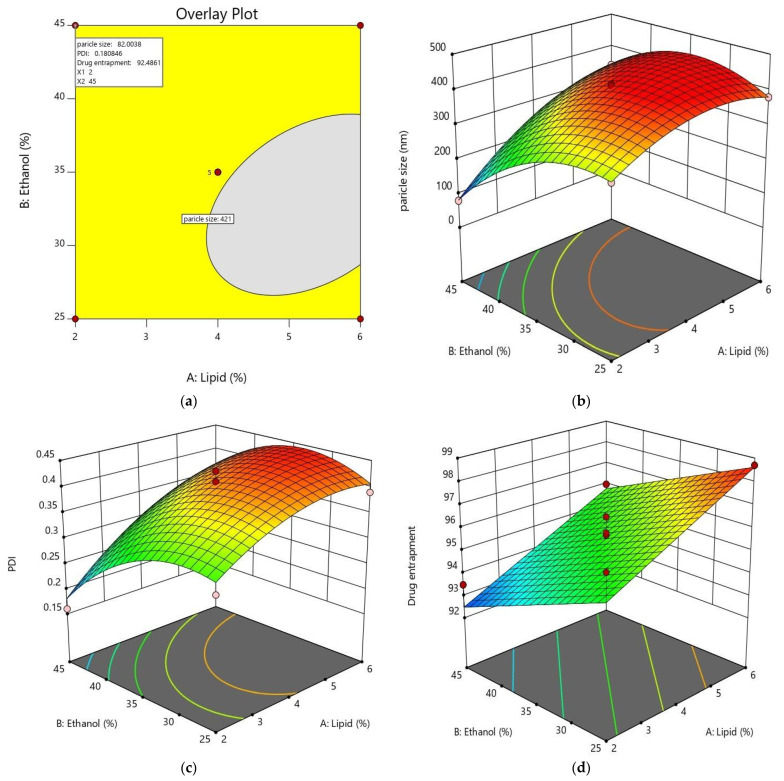
Overlay plot generated by Design of Expert depicting (**a**) a robust yellow region, (**b**) a 3D response surface graph depicting the effect of the independent variable on particle size, (**c**) effect of the independent variable on PDI and (**d**) effect of the independent variable on drug entrapment.

Next and the last step in the optimization process involves the identification of a region (yellow region) in the design space (Figure 4a), through which a robust formulation can be developed. The identified region can suggest a formulation for which dependent responses can be predicted accurately. For this, the software (Design expert version 11) was programmed with constraints and priorities for dependent and independent variables (Table 4) and an overlay plot was generated, in which, a yellow region was identified (Figure 4a). Further, as suggested by this region, when a formulation was prepared and particle size and drug entrapment were assessed, the observed values were almost equal to the predicted values. Therefore, it can be ascertained that this model gives the best fit and it is validated.

The above study outcome provides support and understanding for the preliminary role of the identified parameters and QAs. These are critical to the formulation and any alteration will pose serious threats. These attributes are tabulated alongside their reason for criticality in Table 5 [42].

### 3.4. Particle Size and Polydispersity Index (PDI)

The particle size of the TEF-loaded ethosomes was calculated by the DLS technique and tabulated in Table 6. The effect of lipid and ethanol concentrations was studied on particle size and PDI. It was seen that the concentration of lipids has a constructive effect on vesicular size which means if the lipid amount increases then the size of the vesicles also increases. Further, vesicular size varies inversely with ethanol concentration which means if the concentration of ethanol increases then vesicular size decreases as shown in Figure 4b,c. ANOVA suggests that the model is quadratic in nature having R^2^ values of 0.9996 and 0.9307 for particle size and PDI respectively with a significant F-value for the model and a non-significant *p*-value for the lack of fit.

It was noted that the observed particle size (84 ± 1.88 nm) and PDI values (0.128 ± 0.03) were close to the suggested values. For all formulations, the PDI, which is a measure of the size distribution of the ethosomes, was within the suggested range.

### 3.5. Entrapment Efficiency

Determination of entrapment efficiency is imperative to ensure efficient drug delivery. For all the suggested combinations, entrapment efficiency was calculated and enlisted in Table 6. It was noticed that by increasing the amount of lipid entrapment efficiency increases, whereas increasing the concentration of ethanol drug entrapment decreases as shown in Figure 4d. In this case, also, ANOVA suggests that the model is quadratic in nature having an R^2^ value of 0.8443 with a significant F-value for the model and a non-significant P value for the lack of fit [43].

It was noted that the observed values of entrapment efficiency (94.5% ± 1.54%) are near the suggested values. The high entrapment may be ascribed to the lipophilic nature of the TEF along with better compatibility between TEF and lipids (lipoid S 100).

### 3.6. Characterization of Ethosomes

#### 3.6.1. Morphology of the Ethosomal Formulation with Transmission Electron Microscopy (TEM)

**Figure 5:**
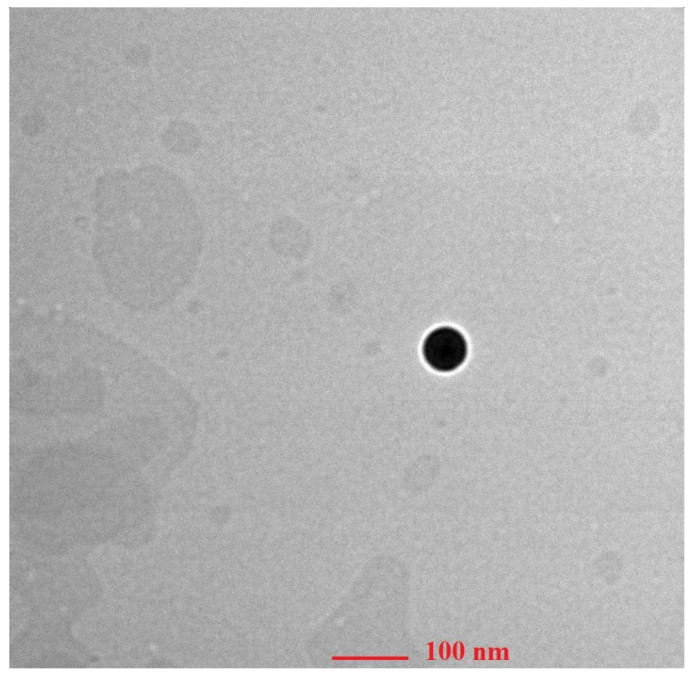
TEM images of ethosomal vesicles depicting size below 100 nm.

Figure 5 shows the TEM images of the TEF-loaded ethosomes. The TEF was entrapped in the core of the vesicles appears darker. The vesicular size was found to be below 100 nm which is within our required size range.

#### 3.6.2. Zeta Potential

One of the major factors influencing formulation storage stability is zeta potential, therefore it was calculated and was observed to be −21 ± 1 mV, indicating good stability of the ethosomes. The higher negative value of zeta potential indicates repulsive interaction between vesicles, and thus prevents accumulation of vesicles. Further, the negative value of the zeta potential might be owing to the presence of Lipoid S 100 in the formulation.

### 3.7. Stability Studies

The size, PDI, and drug content of ethosomal formulation stored at 4 °C for four weeks were investigated, and the results are summarised in Table 7. Ethosomes were found to be fairly stable, as there was no increase in size or PDI, nor there a decrease in drug content. This implies that, under appropriate storage conditions, ethosomal formulations were able to maintain their size without any drug leakage or leaching [44].

### 3.8. Evaluation of TEF-Loaded Ethosomal Gel

#### 3.8.1. Homogeneity

The optimized TEF gel revealed adequate homogeneity with no evidence of grittiness.

#### 3.8.2. Drug Content

In pharmaceutical formulation, high drug content is a desirable characteristic. The drug (TEF) content of the TEF-loaded ethosomal gel was found to be 94.3% ± 1.54%, supporting the choice of gelling agent.

### 3.9. Rheology

**Figure 6:**
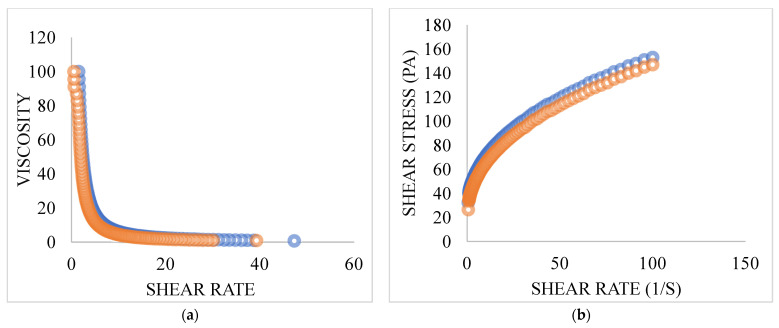
Gel rheological measurements with the help of a rheometer at 25 °C with variations in the (**a**) viscosity; (**b**) shear stress with respect to change in the shear rate. (Orange colour: Ethosmal gel, Blue colour: Conventional gel).

The viscosity of formulated TEF-loaded conventional gel and ethosomal gel were found to be 47.3 Pa.s and 45.9 Pa.s respectively. The changes in viscosity and shear stress as a function of applied shear rate for both conventional gel and ethosomal gel were taken and shown in Figure 6. From the below-mentioned picture, it is clear that with the alteration of shear rate from 0.1 s^−1^ to 100 s^−1^, the viscosities of both the formulations decreased which implied that both gels acted as shear thinning (non-Newtonian) pseudoplastic fluids. As a result, the flow behaviour can be managed at reduced energy owing to the decreased viscosities at a higher shear rate, and the gels were shown to have good thixotropy properties, which offered industrial applications in the future. Further, when the gels were sheared, the network of polymers was broken down and microscopic layers of gel slides onto the neighbouring layer, indicating the increase of the shear stress. These plots represent the gel’s shear-thinning behaviour as a whole [45].

### 3.10. Texture Analysis

**Figure 7:**
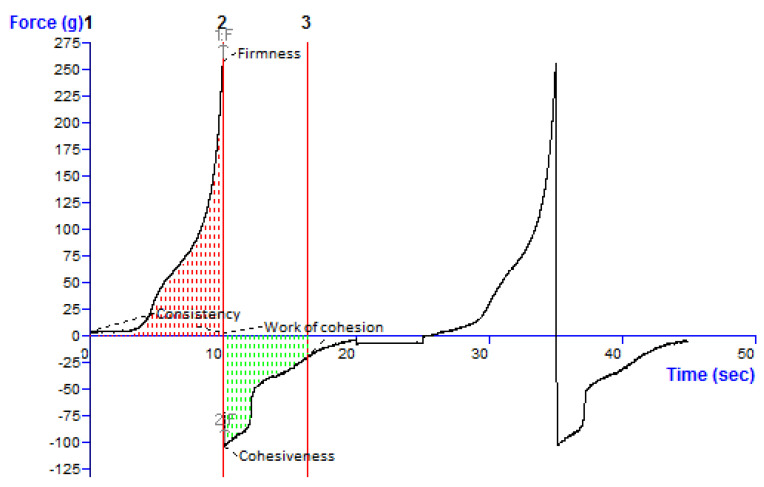
Texture analysis data of the TEFloaded ethosomal gel. The force-time plot was used to calculate parameters such as firmness, consistency, cohesiveness, and work of cohesion. The maximum positive force represents the hardness/firmness of the gel whereas cohesiveness is described as the negative area under the force-time curve.

Other rheological features, such as firmness, consistency, cohesiveness and work of cohesion were additionally described through textural profile evaluation (Table 8). As there is no significant difference in viscosities of both the types of gel hence only ethosomal gel was taken for texture analysis. The texture curve (Figure 7) demonstrates uniformity, the absence of grittiness/lumps, and the smoothness of the gels. The gel showed quite good gel strength, and consistency, which affects the diffusibility and extrudability from the container tube. The gel displayed a satisfactory cohesiveness which is a pre-requisite for adherence to the formulation at the site of application. Hence, the developed gel qualifies as a suitable carrier for the chosen fraction of the CR (TEF).

### 3.11. In Vitro Drug Release Study

**Figure 8:**
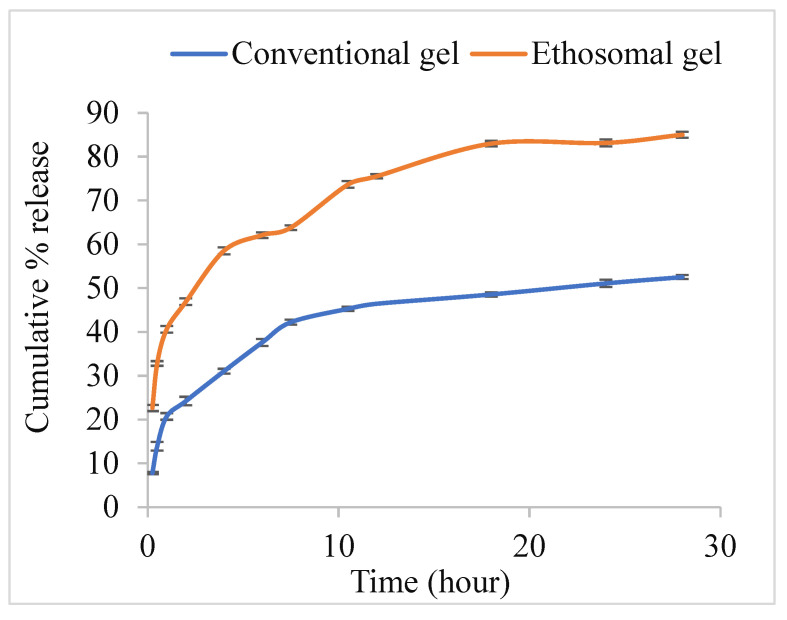
In vitro release study profile of TEF-loaded conventional gel and TEF-loaded ethosomal gel. Each study was performed in triplicate and data are shown as mean ± SD. A comparison of the release profile of both gels is shown with an initial burst release followed by a sustained release pattern.

This study is an essential pointer of the success of a formulation in terms of drug delivery (here it is TEF) via a chosen carrier system. Aliquots of release media were withdrawn at predetermined time intervals ranging from 0 to 28 h and analysed at a specific wavelength against a blank (pure release media) to determine the cumulative amount of the TEF. Drug release rates for both conventional gel and ethosomal gel were calculated, and plots were drawn between the time interval and cumulative percent of drug released. The plots (Figure 8) show that conventional gel and ethosomal gel have an initial burst release of 7.8% and 22.7%, respectively, followed by a slower and sustained release. This biphasic release pattern may possibly be due to the initial rapid release of adsorbed TEF present on the surface followed by the diffusion of TEF through the lipid layer of ethosomes that provides hindrance causing a decrease in cumulative release rate. This release profile with the initial fast release for maintaining a high-concentration gradient is essential for effective transdermal drug delivery to the blood. Further, the cumulative rate of release of TEF from the conventional gel and ethosomal gel was found to be 51% and 83% at 24 h. This higher rate of release of ethosomal gel in comparison to conventional gel may be attributed to the incorporation of ethosomal vesicles in the gel matrix that facilitates easier penetration through the gel matrix. Additionally, the release kinetics of the formulation followed the Peppas model, having an R^2^ value of 0.933 and 0.950 with a corroborating release profile obeying Fickian diffusion for conventional gel and ethosomal gel respectively [46,47]. Further, different release kinetic modelling for ethosomal gel such as zero-order, first-order, Higuchi, and Korsmeyer Peppas was described in Figure S1 to support the analysis.

### 3.12. Ex Vivo Skin Permeation Study

**Figure 9:**
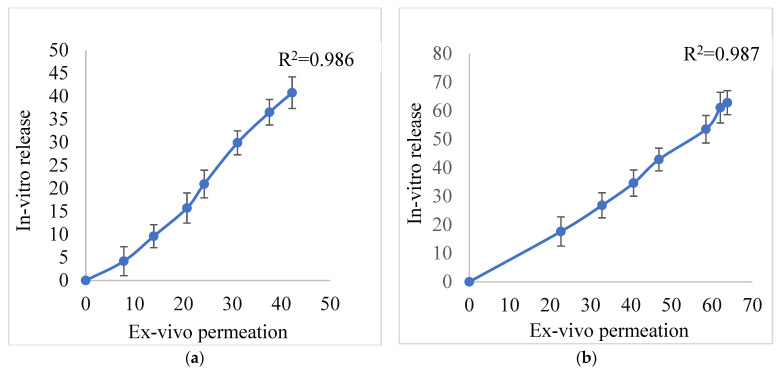
Correlation between in vitro release and ex vivo permeation studies for (**a**) TEF-loaded conventional gel and (**b**) TEF-loaded ethosomal gel. The R^2^ value in plots revealed a close linear correlation between in vitro release and ex vivo permeation data in conventional gel a well as in ethosomal gel.

The skin permeation of the formulation was evaluated using Franz diffusion cells. The permeability of formulation into rat skin was revealed by sampling from 0 to 8 h (clinical application time). The flux of conventional gel and ethosomal gel were found to be 4.182 µg/cm^2^/h and 6.771 µg/cm^2^/h respectively. The permeation coefficients for conventional gel and ethosomal gel were found to be 0.398 and 0.644 cm^2^/h, respectively. The above results show that ethosomal gel has greater permeability than conventional gel. This could be attributed to the ultra-flexible nature of the ethosomes. Figure 9 depicts a linear relationship between in vitro release and ex vivo permeation studies for both conventional gel and ethosomal gel. The R^2^ value in plots evidently revealed a close linear correlation between studies.

### 3.13. Skin Permeation Dynamics

#### 3.13.1. DSC Analysis

**Figure 10:**
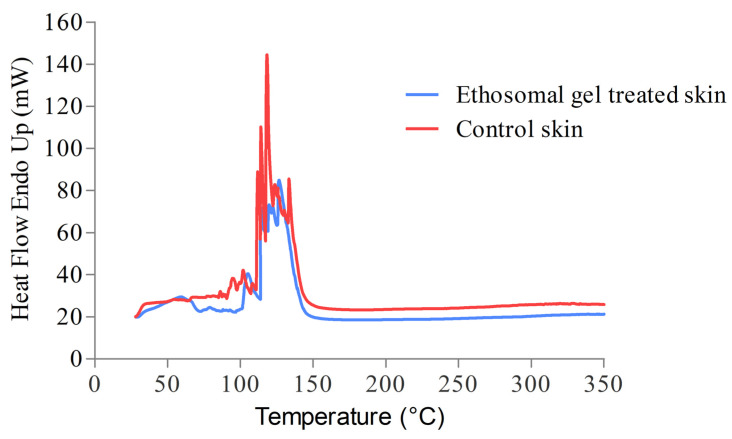
Comparative DSC thermogram between the control skin and ethosomal gel treated skin showing exothermic peaks corresponding to the phase transition of lipids present in the skin.

DSC is a useful tool to obtain information regarding lipid and protein components of the animal skin treated with formulation. The exothermic peaks were acquired in the DSC thermogram for both the un-treated (control skin) and treated skin (with ethosomal gel) as displayed in Figure 10. In both untreated and treated stratum corneum, an exothermic peak corresponding to the phase transition of constituent lipids was observed at 101.7200 °C, 113.359 °C, 116.5090 °C, and 132.855 °C. Nevertheless, there was a decrease in the height and area of the peaks with the treated skin. The results showed that ethosomal formulation treatment slightly disrupted the structures of lipid bilayers in the stratum corneum [48]. This disruption in no way causes any anatomical or physiological changes however supports the accentuated permeability of the ethosomal gel and would correspond to better drug delivery across the skin barrier.

#### 3.13.2. FTIR Analysis

**Figure 11:**
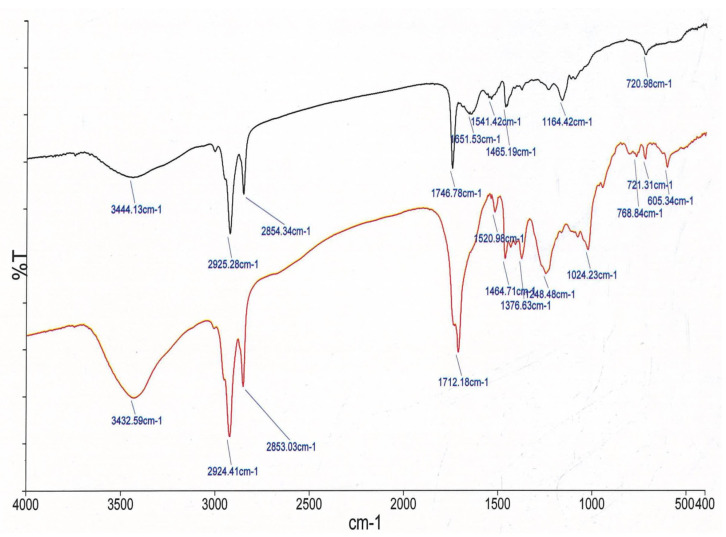
Comparative FTIR spectra between control skin and ethosomal gel-treated skin showing several peaks signify the molecular vibration of lipids and proteins present in the stratum corneum. (Red—Control skin, Black—Ethosomal gel treated skin).

Garidel et al. [49] have suggested that FTIR could be used as a tool to ascertain the permeation dynamics through treated skin (ethosomal gel) and un-treated skin (control) through an informed comparison between various C-O stretching, C-H stretching, and C-N stretching. These peaks signify the molecular vibration of lipids and proteins in the stratum corneum (SC). As a matter of fact, it contains ceramides, cholesterol and fatty acids as its essential components which correspond to the aforementioned characteristic peaks. The characteristic peaks of untreated skin were owing to asymmetric C-H stretching of lipids and proteins and appeared at 2924.41 cm^−1^ while the symmetric C-H stretching is visualized at 2853.03 cm^−1^. The peaks at 1376.63 cm, 1712.18 cm^−1^, and 1464.71 cm^−1^ were caused because of C-N stretching (amide-II), C=O stretching vibrations (amide-I), and CH_3_ bending stretching vibration (protein) respectively. The appearance of these peaks clearly indicates the presence of protein (keratin) in the stratum corneum [50]. The component bands of amide II reflect the secondary structures of keratin. The area and height of these peaks are the reflections of the amount of the lipid in the stratum corneum. Therefore, any variation in the area, height, wave number and intensity of the CH_2_ stretching peaks can reflect the alteration in the strength of lipids in the stratum corneum layer. The presence of an ethosomal vesicle appears to have distorted the lipid bilayer, as evidenced by a shift in CH_2_ stretching peaks to higher wavenumbers. This could be translated as a success of the scientifically crafted ethosomal gel which could transcend the stratum corneum barrier properties. Stratum corneum is also embedded with tightly packed lipids (ceramides, amide I and amide II) as bilayers which contributed to strong hydrogen bonding and was perhaps accountable for the barrier property of the stratum corneum hence it becomes the rate-limiting factor in transdermal drug delivery [51,52]. When the skin was treated with ethosomal gel, the hydrogen bonding network at the ceramide surface broke down, indicating permeation of formulation through the lipidic double layers of the stratum corneum. This can be seen from Figure 11 where there is a shifting of peaks of 2924.41 cm^−1^ due to symmetric C-H stretching 2853.03 cm^−1^ to the higher wavenumber and also with alteration in area, height and intensity as compared to untreated skin. In FTIR spectra, the corresponding peaks to C-N and C-O stretching appeared with an increase in wavenumber and dimension when compared to untreated skin [48].

As a result of this FTIR study, we can conclude that lipid disruption and fluidization, as well as protein denaturation, are the primary causes of increased skin permeation.

#### 3.13.3. Histological Investigation of the Skin

**Figure 12:**
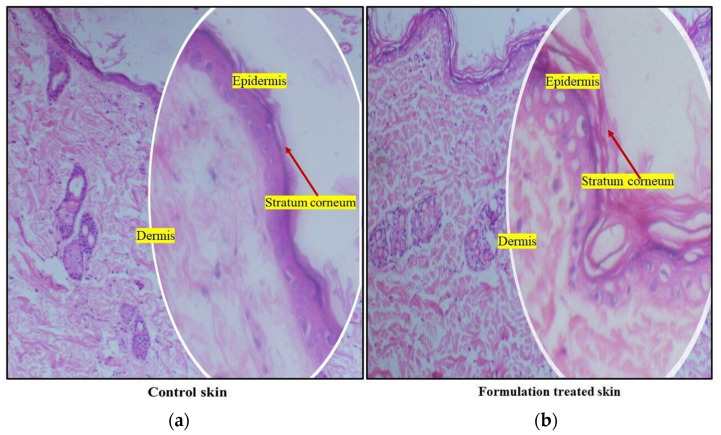
Comparison of histological cross sections of the (**a**) Control skin and (**b**) treated skin (6 h treatment with ethosomal gel) (Magnification; 10× and 40× (within circle).

Cross sections of the skins (both control and ethosomal gel treated) were prepared and observed microscopically. The images of the control skin showed an intact SC with well-defined margins along with the SC, epidermis, and dermis (Figure 12a ). The dermis did not show any evidence of inflamed cells. The skin appendages were regular, and the collagen fibre bundles were tightly packed. However, after 6 h, the SC in the ethosomal gel-treated skin showed clear morphological changes (Figure 12b). The SC had a compromised surface eliciting randomly interwoven layers in the formulation-treated skin. This could be due to the presence of surfactants, which are reportedly known to extract intercellular lipids and disrupt connections between keratinocyte desmosomes [53,54] or else could be attributed to the movement of ultra-flexible ethosomal vesicles, thus enhancing the lipid bilayer interlamellar volume in the SC. Furthermore, ethanol a major component of ethosomes is an established permeation enhancer that supposedly manoeuvres the skin lipid bilayer organization thus affecting the intercellular region of the SC [55]. The presence of formulation in the epidermis increased the intercellular spaces and distinct voids. The collagenous bundles in the dermis were separated abnormally. This finding suggests that ethosomes were escaping from gels, disrupting the macroscopic structure of the skin barrier and extracting intercellular lipids. This could account for the histological changes observed in this study. Further, the histological examinations revealed no inflamed cells in the dermis, indicating that the ethosomal gel was topically safe.

#### 3.13.4. Assessment of Depth of Skin Permeation

**Figure 13:**
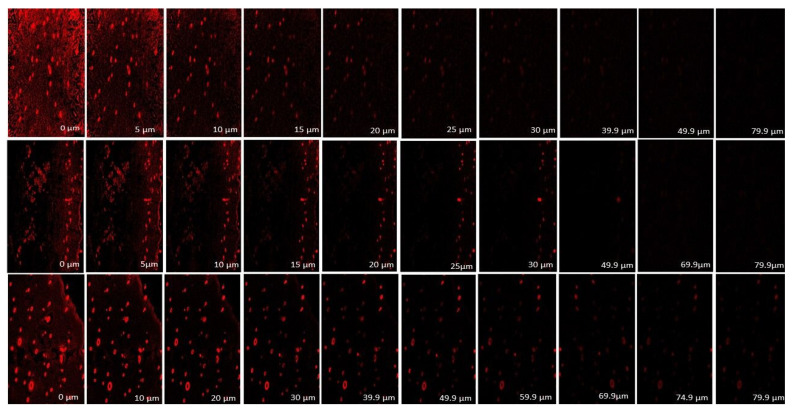
CLSM image of the skin showing skin permeation of the rhodamine B from rhodamine B solution (1st row), rhodamine B-loaded conventional gel (2nd row) and rhodamine B-loaded ethosomal gel (3rd row). The red colour indicates the fluorescence intensity produced by the rhodamine B dyes entrapped inside the skin layers.

**Figure 14:**
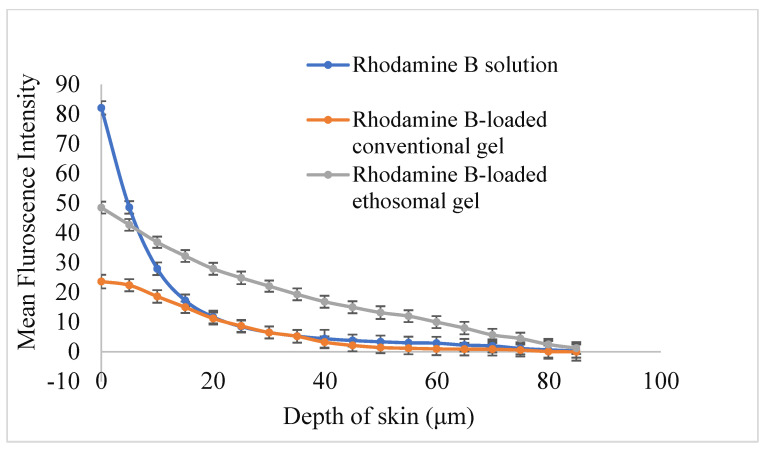
Comparative CLSM plots between the mean fluorescence intensity produce by rhodamine dye and depth of skin permeation after treating the skin with rhodamine B solution, rhodamine B-loaded conventional gel and rhodamine B-loaded ethosomal gel.

To determine the skin permeation strength, a comparative CLSM analysis was performed between rhodamine B solution, rhodamine B-loaded conventional gel, and rhodamine B-loaded ethosomal gel up to a depth of 80 μm. The confocal images (Figure 13) illustrated the intensity of fluorescence in skin treated with rhodamine solution, conventional gel and ethosomal gel, respectively, at various depths. All the samples displayed an intracellular distribution. In case of the solution-treated skin sample, the fluorescence intensity in the skin was found very high immediately after application as it was solubilised in a hydro-alcoholic mixture so the influence of ethanol may improve penetration. But gradually there was a sharp decrease in intensity and became insignificant after 40.0 µm. In the case of conventional gel, the fluorescence intensity in the skin was initially higher (but very less as compared to the dye solution) and might be attributed to the burst release behaviour of the formulation and then followed a sustained action and the depth of penetration was found up to 55 µm. In case of ethosomal gel, the initial fluorescence intensity and depth of penetration both were comparatively more demonstrating the superiority of the ethosomal formulation, which could be ascribed to the presence of flexible vesicles within the gel. Figure 14 shows a graphical representation of the comparative skin permeation of the rhodamine B from rhodamine B solution, rhodamine B-loaded conventional gel and rhodamine B-loaded ethosomal gel through animal skin. The depth of skin permeation analysis proved that ethosomal gel followed deeper penetration of the TEF by crossing the impermeable epidermal layer of skin. Hence, this ethosomal gel incorporating TEF can be proposed for transdermal application [56].

### 3.14. Skin Irritation Studies

To ensure the suitability of formulation for application on the skin this study as carried out. The study was evaluated based on the skin erythema and oedema, produced at the application site, which reflects the skin-irritating ability of the formulation. Even after 3 days, neither the TEF solution nor the optimised TEF-loaded ethosomal gel caused any irritation at the application site. The scores we zero hence signifying the non-irritant nature of both the TEF solution and the TEF-loaded ethosomal gel [57]. These findings corroborated the findings of a histological examination of the skin following treatment with an ethosomal gel.

## 4. Conclusions

A newer formulation premise of ethosomal gel as a carrier for CR-TEF has been developed which would circumvent the skin barriers for better dermal delivery and consequently mitigate the symptoms associated with menopause. The scientifically crafted ethosomal formulation promises an adequate drug entrapment into lipophilic vesicles assisted with the presence of ethanol which bestows ultra flexibility and ease of permeation to the intact stratum corneum. The pharmaceutical attributes of the optimized formulation were collated by using various quality tools leading to a dependable formulation having small vesicle size, good PDI, improved transdermal flux, better entrapment efficiency with reduced skin irritancy and higher stability at 4 °C. Experimental tools like DSC, IR, histology investigation and CLSM supported the presence of an appreciable permeation of the ethosomal gel. Notably, the vesicular size, PDI and entrapment efficiency were significantly affected by alterations in lipid and ethanol concentrations in formulations as suggested by QbD optimization. Thus, the current research proposal encapsulated a highly feasible QbD, risk assessment-based approach of fabricating TEF-loaded ethosomal gel for menopause.

## Figures and Tables

**Table 1:** Elements of quality target product profile for ethosomal gel. The targets and justification are discussed for each quality target product profile element along with the identification of critical quality attributes. It specifies the desired quality standards of the product.

QTPP Elements	Targets	CQAs	Justification
Dosage form	Ethosomal Gel	-	Semisolid dosage form for ↑ the patient compliance
Route of administration	Transdermal	-	Permeate through the skin without systemic side effects
Dosage strength	Good	-	Influence the frequency of application
Vesicular size	Small (˂100 nm)	yes	Affect the drug permeation, drug release & content uniformity
PDI	Uniform	yes	Affect the drug permeation, drug release & content uniformity
Ethanol concentration	˂50%	yes	Affect the vesicular size, PDI, drug entrapment & skin irritation
Lipid concentration	Optimized	yes	Influence the vesicular size, PDI & drug entrapment
Surfactant concentration of	Minimum	yes	Affect the vesicular size, PDI, drug entrapment & stability of the formulation
pH	Compatible with skin	Yes	Affect the physiochemical stability & irritability
Temperature	Optimized	Yes	Impacts the vesicular size & PDI
Solubility	-	Yes	Impacts the formulation process & drug permeation
Log P	-	Yes	Impacts the drug release, skin permeation & retention
Drug entrapment	High	Yes	Affecting drug delivery, dermal dissemination & dosing frequency
Drug release	Good	Yes	Affect the formulation success
Needle type	Small size	Yes	Impacts the vesicular size & PDI
Injection rate	Optimized	Yes	Affects the vesicular size & PDI
Stirring speed	Optimized	Yes	Impacts the vesicular size & PDI
Stirring time	Optimized	Yes	Influences the vesicular size & PDI

Note. ↑: Increase.

**Table 2:** Optimized values of fixed parameters for formulation. Process variables such as time of stirring, speed of stirring, temperature and surfactant concentration were fixed on the basis of the outcome of formulation trials carried out for initial risk assessment.

Parameter	Optimized Value
Stirring time	10–15 min
Stirring Speed	2200–2300 rpm
Temperature	30°
Concentration of Surfactant	1%

**Table 3:** Different factors and their ranges in Design Expert software with constraints for dependent variables.

Factors	Levels and Ranges		
	−1	0	+1
Concentration of ethanol(% *v*/*v*)	25	35	45
Concentration of lipid (Lipod S 100) (% *v*/*v*)	2	4	6

**Table 4:** Lists of dependent variables and independent variables for the formulation. It also enlists the range of formulation variables used for the optimization of the formulation.

Factors	Coded Levels	
Independent Variables	Low Level (−1)	High Level (+1)
A: Concentration of ethanol (% *v*/*v*)	25	45
B: Concentration of lipid (Lipoid S 100) (% *v*/*v*)	2	6
**Dependent Variables**		**Constraints**
Response 1 = Particle Size (nm) **(Priority set at: +++++)**		80–421
Response 2 = PDI **(Priority set at: +++)**		0.16–0.45
Drug Entrapment **(Priority set at: +++++)**		94–98.5

**Table 5:** Depicts the critical quality attributes and reason for their criticality for the preparation of ethosomal formulation.

Quality Attributes (Range of Acceptance)	Reason for Criticality
Particle size (≤200 nm)	Good Skin Penetrability
PDI (≤0.3)	Size Uniformity
% Drug Entrapment (100)	Maximum drug utilization

**Table 6:** Formulation runs with dependent variable responses. These are generated by Design of Expert depicting predicted particle size, PDI and drug entrapment.

S No.	Formulation Code	Lipid (% *w*/*v*)	Ethanol (% *v*/*v*)	Particle Size (nm) Mean ± SD (*n* = 3)	PDI Mean ± SD (*n* = 3)	Drug Entrapment (%) Mean ± SD (*n* = 3)
1	F 01	4	35	413 ± 2.08	0.41 ± 0.04	95.8 ± 0.75
2	F 02	6.82843	35	421 ± 2.08	0.45 ± 0.07	98 ± 1.81
**3**	**F 03**	**2**	**45**	**80 ± 2.08**	**0.16 ± 0.03**	**93.5 ± 1.74**
4	F 04	4	35	416 ± 1.52	0.43 ± 0.02	96.5 ± 2
5	F 05	6	45	342 ± 2.08	0.35 ± 0.05	96 ± 2.3
6	F 06	6	25	380 ± 3.05	0.39 ± 0.05	98.7 ± 2.8
7	F 07	2	25	310 ± 2.64	0.3 ± 0.06	96.5 ± 2.2
8	F 08	1.17157	35	188 ± 1.52	0.268 ± 0.04	92 ± 2.7
9	F 09	4	20.8579	350 ± 3.78	0.38 ± 0.06	97 ± 4
10	F 010	4	49.1421	163 ± 1.15	0.25 ± 0.07	93 ± 2.6
11	F 011	4	35	408 ± 4.04	0.36 ± 0.03	95 ± 2.6
12	F 012	4	35	410 ± 2	0.41 ± 0.05	95.7 ± 3.2
13	F 013	4	35	414 ± 2.08	0.43 ± 0.06	94.5 ± 3.5

**Table 7:** Stability parameters.

Parameters	0 Week	1st Week	2nd Week	3rd Week	4th Week
Size	84 ± 1.88	89 ± 3	96 ± 2.4	103 ± 3.30	108 ± 1.98
PDI	0.128 ± 0.03	0.243 ± 0.06	0.286 ± 0.07	0.312 ± 0.01	0.311 ± 0.03
Drug entrapment	94.5% ± 1.54%	94.2% ± 2.1%	93% ± 1.9%	92.12% ± 1.5%	92.2% ± 2.1%

**Table 8:** Texture analysis data depicting the values of firmness, consistency, cohesiveness and work of cohesion of ethosomal gel.

Firmness (g) Force 1	Consistency (g. s) Area F-T 1:2	Cohesiveness (g) Force 2	Work of Cohesion (g. s) Area F-T2:3
257.93	530.31	−104.30	−349.20

## Data Availability

Not applicable.

## Supplementary Materials

The following supporting information can be downloaded at: https://www.mdpi.com/article/10.3390/pharmaceutics15020465/s1, Table S1. Possible failure modes affected by altering CMAs and CPPs derived from CQAs. Here, each CQAs has been examined with respect to all the related CMAs and CPPs. It further discusses how these CMAs and CPPs affect the CQAs resulting in the failure of the formulation, Table S2. FMEA of the material attributes and process parameters involved in the preparation of the formulation along with their failure effects, cause and controls in quantifying risk. The attribute of failure modes of critical material attributes and critical process parameters are classified into high, medium and low risks. These risks were quantified basis on their failure effect, potential cause and control established., Table S3. Scoring table designed by the formulator considering the severity, probability of occurrence and probability of detectability of failure mode of the critical quality attributes associated with the formulation. The score was devised in the range of 1–10, Table S4. Risk-level rating criteria. The risks were denoted as low, medium and high according to the risk priority number. It gives a realistic picture with a correlation of all variables related to process and materials with scrupulous importance to CQAs resulting in precise quantification of threat during the formulation process., Table S5. Formulation trials for initial risk assessment. It describes how the concentration of lipid, ethanol, and surfactant; temperature; time of stirring; the speed of stirring affect the particle size and PDI of the formulation. Figure S1. Different release kinetic modelling to support the release analysis (a) Higuchi matrix (b) Zero order kinetics (c) Korsemeyer Peppas (d) First order kinetics.
