# Supplementary material for: Quality by Design Assisted Optimization and Risk Assessment of Black Cohosh Loaded Ethosomal Gel for Menopause: Investigating Different Formulation and Process Variables
Source: Pharmaceutics. 2023 Jan 31;15(2):465. doi: 10.3390/pharmaceutics15020465 (PMC9966456; doi:10.3390/pharmaceutics15020465)
Supplement: Supplementary file 1 [file pharmaceutics-15-00465-s001.zip › pharmaceutics-2020900-supplementary.pdf]

pharmaceutics

# Supplementary Materials: Quality by Design Assisted Optimization and Risk Assessment of Black Cohosh Loaded Ethosomal Gel for Menopause: Investigating Different Formulation and Process Variables

**Sradhanjali Mohapatra, Mohd. Aamir Mirza, Sayeed Ahmed, Uzma Farooq, Mohammad Javed Ansari, Kanchan Kohli and Zeenat Iqbal**

**Table S1.** Possible failure modes affected by altering CMAs and CPPs derived from CQAs. Here, each CQAs has been examined with respect to all the related CMAs and CPPs. It further discusses how these CMAs and CPPs affect the CQAs resulting in the failure of the formulation.

| CQAs | Related to CMAs | Related to CPPs | Failure Mode |
|---|---|---|---|
| Particle size | Change in raw material particle sizes | • Speed of mixing<br>• Time of mixing<br>• Injection rate | Changes in content uniformity, drug release & dermal distribution of the drug |
| PDI | Variation in particle sizes distribution of raw materials | • Speed of mixing<br>• Time of mixing<br>• Injection rate | Changes in content uniformity, drug release & dermal distribution of the drug |
| Ethanol concentration | Change in proportion of ethanol & water | • Process temperature<br>• High duration of mixing | Changes in formulation microstructure, particle size, skin retention, permeation & impact on sensorial attributes of the product |
| Lipid concentration | Change in proportion of lipid & water | Process temperature | Alteration in size, PDI & entrapment |
| Surfactant concentration | Change in proportion of surfactant & other raw materials | Specific concentration is desired | Decrease the stability & alters absorption |
| pH | Amount of TEA in the formulation | Consistent addition of TEA with optimized stirring | Alters ionic strength |
| Temperature | - | Consistent temperature | Non-uniform size & PDI |
| Solubility | Change in raw material | Temperature | Precipitation & phase separation |
| Viscosity (shear stress & shear rate) | • Viscosity of liquid/semisolid raw materials<br>• Concentration of polymer | Speed of mixing<br>Time of mixing | Changes in skin retention of the formulation & drug penetration through the skin Changing in patient acceptability/compliance, Impact on sensorial attributes of the product |
| Content uniformity | - | • Speed of mixing<br>• Time of mixing | Dose Variability |
| Drug entrapment | Changes in lipid & ethanol concentration | • Speed of mixing<br>• Time of mixing | Affecting drug delivery, dermal dissemination & dosing frequency |

| | | | |
|---|---|---|---|
| Drug release | Alteration in lipid & ethanol concentration | • Rotation speed<br>• Temperature of medium | Affecting drug delivery, dermal dissemination & formulation failure |
| Needle type | Alteration in needle size | • Speed of mixing<br>• Time of mixing | Affecting the particle size & PDI |
| Injection rate | Defective plunger | • Speed of injection(plunger pressure)<br>• Speed of mixing<br>• Time of mixing | Inhomogeneous entrapment, size & multiplied PDI |
| Precipitation/aggregation | Dependent on the type of emulsifier, gelling agent or volatiles | • The order of addition<br>• High duration of mixing | Influence on API partitioning within the formulation, Amount of drug permeating through the skin |

**Table S2.** FMEA of the material attributes and process parameters involved in the preparation of the formulation along with their failure effects, cause and controls in quantifying risk. The attribute of failure modes of critical material attributes and critical process parameters are classified into high, medium and low risks. These risks were quantified basis on their failure effect, potential cause and control established.

| Check Points/Inputs | Failure Mode | Failure Effects | S | Potential Cause | O | Controls established | D | RPN | Risk |
|---|---|---|---|---|---|---|---|---|---|
| | | | | **CPP** | | | | | |
| Particle size | Larger particle size | Changes in dose uniformity, drug release & dermal distribution of the drug | 8 | Alterations in the concentration of ethanol, lipid, temperature & stirring speed are key factors affecting the size | 8 | The measured amount of ethanol & lipid will be a decisive effect with control temperature & stirring speed | 7 | 448 | High |
| PDI | Non Uniformity | Changes in dose uniformity, drug release & dermal distribution of the drug | 5 | Alterations in the concentration of ethanol, lipid, temperature & stirring speed are key factors affecting the size | 6 | The measured amount of ethanol & lipid will be a decisive effect with control temperature & stirring speed | 5 | 150 | Medium |
| Drug entrapment | Affects drug release | Varying minimum effective concentration | 8 | Solubility, amount of lipid & ethanol | 8 | Optimized amount of ethanol & lipid | 6 | 384 | High |
| Drug release | Changes in effectiveness /pharmacological action | Formulation failure | 9 | Improper drug entrapment & drug loading | 6 | Appropriate drug entrapment & loading | 5 | 270 | Medium |
| Content uniformity | Changes in dose uniformity | Variable dosing & drug release from the formulation | 5 | Inadequate solubility, & high PDI | 5 | Low PDI with suitable excipients | 4 | 100 | Low |
| Ethanol concentration | Change in particle size and PDI | Changes in the size of the formulation, PDI, drug | 9 | Contributes majorly to drug & lipid solubility. Further, it | 8 | Optimized quantity of ethanol is critical for parameters like | 9 | 648 | High |

| | | | | | | | | | |
|---|---|---|---|---|---|---|---|---|---|
| | | entrapment, skin retention and permeation of the active ingredient | | acts as a penetration enhancer hence, alteration of its concentration has a marked effect on drug penetration & size of the particles | | particle size, PDI, drug entrapment, drug penetration & drug loading | | | |
| Lipid concentration | Inadequate size, drug entrapment & drug loading capacity | Changes in the size of the formulation, drug entrapment & drug loading | 9 | Lipid concentration contributes majorly to formulation thereby affecting size, drug entrapment & loading capacity | 8 | An accurate quantity of lipid concentration is crucial for related parameters | 8 | 576 | High |
| Surfactant concentration | Instability | Alteration in size, PDI & stability of the formulation | 2 | Contributes majorly to formulation stability | 3 | Optimized concentration of the surfactant concentration is essential for formulation stability | 3 | 18 | Low |
| Solubility | Variation in drug content, drug loading, particle size & PDI | Differing amounts of drug concentration thereby resulting in assorted drug loading, particle size and PDI | 7 | Solubility is affected by solvents & the amount meant for solubilising the active ingredient & excipients to uniformity. Improper solvents mean non-uniform solubility | 6 | Proper selection of excipients | 4 | 168 | Medium |
| Log P | Inadequate permeation | Changes in skin permeation & skin physiology | 3 | Inherent properties of the active ingredient | 3 | | 3 | 27 | Low |
| Viscosity, shear stress and shear rate | ▪ Changes in skin adhesion & permeation ▪ Extrusion from the container | ▪ Alteration in skin adhesion of the formulation & followed by drug penetration through the skin ▪ Impact on sensorial attributes of the product ▪ Affect patient compliance | 5 | Inadequate gelling agent or dilution | 6 | Optimized amount of gelling agent | 3 | 90 | Low |
| **CMA** | | | | | | | | | |
| pH | Skin irritation | Variation in drug release & skin irritation | 2 | Improper mass ratio leads to incomplete conversion | 3 | Definite PH is required to avoid skin irritation & drug release | 2 | 12 | Low |
| Temperature | Affects lipid solubility | Alteration in size & PDI of the | 1 | At lower temperature size | 2 | Temperature is heavily monitored & | 2 | 4 | Low |

| | | | | | | | | | |
|---|---|---|---|---|---|---|---|---|---|
| | | formulation. Hence drug entrapment, loading & drug release | | and PDI of the formulation increase | | fixed to the desired level | | | |
| Needle type | Variation in size of the formulation & PDI | Improper particle size & PDI hence causing variation in dose uniformity | 2 | Variation in needle type | 3 | Proper selection of needle | 2 | 12 | Low |
| Injection rate/ Flow rate | Changes in size of the formulation & PDI | Improper particle size & PDI hence causing variation in dose uniformity & coagulation of particles | 3 | Non-uniform flow rate | 3 | Optimized flow rate | 2 | 18 | Low |
| Stirring speed | Alteration in size & PDI of the formulation | Improper particle size & PDI hence causing variation in dose uniformity & coagulation of particles further, it affect the drug entrapment & loading | 7 | Improper speed of stirring | 6 | Maintaining proper stirring speed | 5 | 210 | Medium |
| Stirring time | Alteration in size & PDI of the formulation | Improper particle size & PDI hence causing variation in dose uniformity & coagulation of particles further, it affects the drug entrapment & loading | 3 | Improper speed of stirring | 4 | Maintaining proper stirring speed | 3 | 36 | Low |

**Table S3.** Scoring table designed by the formulator considering the severity, probability of occurrence and probability of detectability of failure mode of the critical quality attributes associated with the formulation. The score was devised in the range of 1-10.

| Score | Severity | Probability of Occurrence | Probability of Detectability |
|---|---|---|---|
| 1 | No Effect | No documented probability of failures | Controls in place to detect the fault |
| 2-4 | Minor-Limited part is affected | Low probability of failures to take place | Low probability that the fault will get undetected |
| 5-7 | Medium | Occasional probability of failures to take place and will have moderate impact on associated parameters | Moderate probability that the fault will get undetected |
| 8-10 | High | High probability of failures to take place and will have a high impact on associated parameters | High probability that the fault will get undetected |

**Table S4.** Risk-level rating criteria. The risks were denoted as low, medium and high according to the risk priority number. It gives a realistic picture with a correlation of all variables related to process and materials with scrupulous importance to CQAs resulting in precise quantification of threat during the formulation process.

| Risk priority number (RPN) | Risks level | Rationale |
|---|---|---|
| 1-100 | Low | The process is robust or controls are in place to handle any change |
| 101-350 | Medium | Process parameters & associated controls are comparatively stout & are capable enough to withstand moderate outcome |
| 351-1000 | High | Any alteration to process parameters/attributes will have serious outcome |

**Table S5.** Formulation trials for initial risk assessment. It describes how the concentration of lipid, ethanol, and surfactant; temperature; time of stirring; the speed of stirring affect the particle size and PDI of the formulation.

| Formulation code | Parameter | Particle size (nm) | PDI |
|---|---|---|---|
| Concentration of Ethanol | | | |
| N1 | 30 % | 405 | 0.52 |
| N2 | 40 % | 188 | 0.30 |
| N3 | 45 % | 85 | 0.21 |
| Concentration of Lipid (Lipoid S 100) | | | |
| N4 | 2 % | 83 | 0.18 |
| N5 | 3 % | 235 | 0.35 |
| N6 | 4 % | 445 | 0.44 |
| Concentration of surfactant (Tween 80) | | | |
| N7 | 1% | 84 | 0.16 |
| N8 | 2% | 118 | 0.26 |
| Temperature | | | |
| N9 | 25 | 410 | 0.41 |
| N10 | 30 | 84 | 0.15 |
| N11 | 35 | 82 | 0.19 |
| Stirring time | | | |
| N12 | 5 minutes | 312 | 0.4 |
| N13 | 10 minutes | 82 | 0.13 |
| N14 | 15 minutes | 89 | 0.15 |
| Stirring speed | | | |
| N15 | 1900 rpm | 225 | 0.2 |
| N16 | 2200 rpm | 85 | 0.15 |
| N17 | 2300 | 81 | 0.16 |

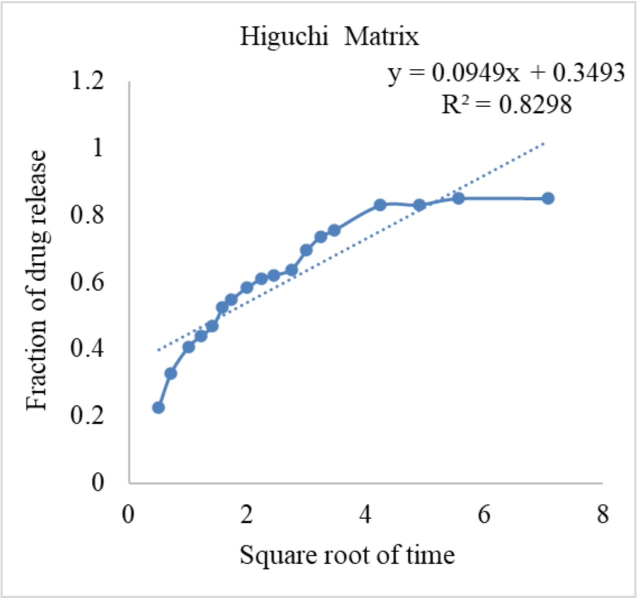


**(a)**

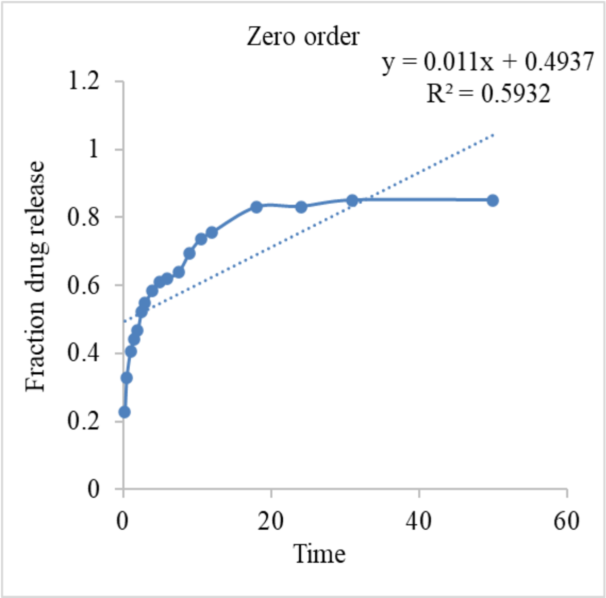


**(b)**

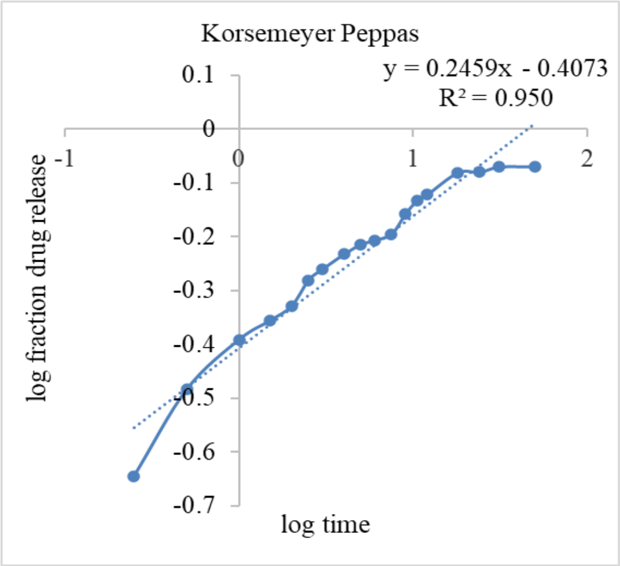


**(c)**

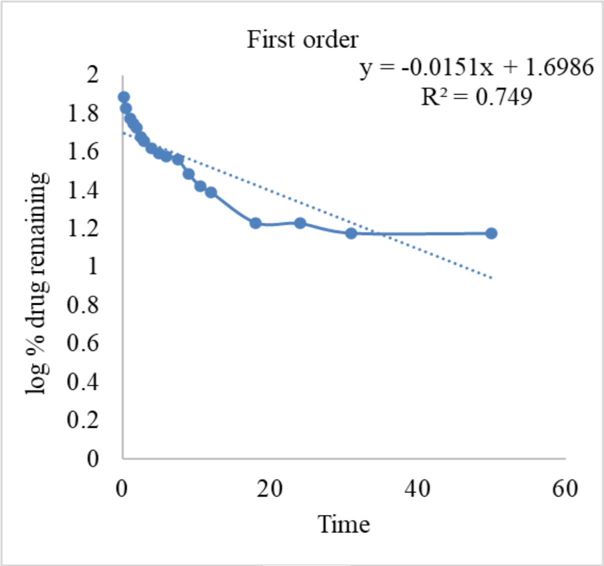


**(d)**

**Figure S1.** Different release kinetic modelling for ethosomal gel to support the release analysis. (**a**) Higuchi matrix (**b**) Zero order kinetics (**c**) Korsemeyer Peppas (**d**) First order kinetics.
